# P28 Assessment of colistin susceptibility in carbapenem-resistant *Klebsiella pneumoniae* and *Escherichia coli*: a comparison of diagnostics MIC-COL and VITEK^®^ 2 AST-XN21 with broth microdilution

**DOI:** 10.1093/jacamr/dlaf230.035

**Published:** 2025-12-04

**Authors:** Fatih Mehmet Akıllı

**Affiliations:** University of Health Sciences Sincan Training and Research Hospital, Ankara, Türkiye

## Abstract

**Background:**

Carbapenem-resistant Enterobacterales (CRE) have been classified by the WHO as a critical priority pathogen. Colistin remains one of the last-resort therapeutic options for treating infections caused by CRE. This study aimed to evaluate the performance of two automated systems—the novel commercial kits Diagnostics MIC-COL (Diagnostics Inc., Galanta, Slovakia) and VITEK^®^ 2 AST-XN21 (bioMérieux, Marcy-l'Étoile, France)—for assessing the in vitro susceptibility of CRE strains to colistin, with broth microdilution (BMD) serving as the reference standard.

**Methods:**

A total of 94 clinical isolates were included, comprising 74 *Klebsiella pneumoniae* and 20 *Escherichia coli* strains, obtained from various clinical specimens collected from hospitalized patients between September 2023 and December 2024. Consecutive isolates were excluded from the study. Isolate identification and antibiotic susceptibility testing were performed using both VITEK and conventional methods. Colistin susceptibility testing was conducted according to the EUCAST criteria, with susceptibility defined as ≤2 mg/L and resistance as >2 mg/L. The categorical agreement (CA), essential agreement (EA), very major error (VME) and major error (ME) rates of the commercial kits were calculated based on BMD results. Quality control strains included *E. coli* ATCC^®^ 25922, *Pseudomonas aeruginosa* ATCC^®^ 27853 and *E. coli* NCTC 13846.

**Results:**

Colistin resistance was identified in 31.9% (30/94) of CRE isolates overall and in 40.5% of carbapenem-resistant *K. pneumoniae* (CR-KP) isolates. The Diagnostics MIC-COL method demonstrated 100% CA, 95.7% EA, with no VMEs or MEs. The VITEK^®^ 2 AST-XN21 method showed 96.8% CA, 89.1% EA, a VME rate of 1.06% (1/94) and an ME rate of 2.1% (2/94) (Table 1).

**Conclusions:**

In conclusion, the high prevalence of colistin resistance among CRE isolates is a significant concern. The findings of this study indicate that both the Diagnostics MIC-COL and VITEK^®^ 2 AST-XN21 systems are reliable alternatives to the reference BMD method for colistin susceptibility testing in CRE isolates. However, further multicentre studies with larger sample sizes, incorporating molecular approaches to detect colistin resistance, are necessary to validate and expand upon these findings.

**Table 1.** Agreement and error rates of commercial methods compared to the reference broth microdilution method

	Diagnostics MIC COL				VITEK AST-XN21			
	CA *n* (%)	EA *n* (%)	VME	ME	CA *n* (%)	EA *n* (%)	VME	ME
*K. pneumoniae* (*n*=74)	74 (100)	68 (91.8)	–	–	71 (95.9)	68 (91.8)	1 (1.3)	2 (2.7)
*E. coli* (*n*=20)	20 (100)	20 (100)	–	–	20 (100)	17 (85)	–	–

